# Additional N-glycosylation mutation in the major hydrophilic region of hepatitis B virus S gene is a risk indicator for hepatocellular carcinoma occurrence in patients with coexistence of HBsAg/anti-HBs

**DOI:** 10.18632/oncotarget.18682

**Published:** 2017-06-27

**Authors:** Yan Qiao, Shanshan Lu, Zhihui Xu, Xiaodong Li, Kai Zhang, Yan Liu, Li Zhao, Rongjuan Chen, Lanlan Si, Shumei Lin, Dongping Xu, Jin Li

**Affiliations:** ^1^ Research Center for Clinical and Translational Medicine/Institute of Infectious Diseases, Beijing 302 Hospital, Beijing 100039, China; ^2^ Clinical Medical School, Guilin Medical University, Guilin 541004, China; ^3^ Medical Department, Beijing 302 Hospital, Beijing 100039, China; ^4^ Department of Infectious Disease, First Affiliated Hospital of Xi’an Jiaotong University, Xi’an 710061, China

**Keywords:** hepatitis B virus, mutation, additional N-glycosylation, HBsAg/anti-HBs coexistence, hepatocellular carcinoma

## Abstract

The study aimed to determine the association of additional N-glycosylation mutations in the major hydrophilic region (MHR) of hepatitis B virus (HBV) S gene with hepatocellular carcinoma (HCC) occurrence in HBsAg/anti-HBs coexistent patients. A total of 288 HBsAg/anti-HBs coexistent patients and 490 single HBsAg-positive patients were enrolled, including 193 with HCC, 433 with chronic hepatitis B (CHB), and 152 with acute-on-chronic liver failure (ACLF). The HBV S genes were amplified from serum and sequenced. The frequency of additional N-glycosylation mutations was significantly higher in HCC patients (12.37%) than in CHB patients (4.39%) and ACLF patients (2.63%). The frequency escalated by an order of single HBsAg-positive non-HCC (1.61%), single HBsAg-positive HCC (5.98%), HBsAg/anti-HBs coexistent non-HCC (8.01%), and HBsAg/anti-HBs coexistent HCC (22.36%). Twelve kinds of mutations/mutation patterns were detected, five of which have not been reported. Multivariate analysis showed that age > 40 years [*OR*, 3.005; 95% CI, 1.177−7.674; *P* = 0.021], alpha-fetoprotein > 10 ng/mL [*OR*, 4.718; 95% CI, 2.406−9.251; *P* <0.001], cirrhosis [*OR*, 6.844; 95% CI, 2.773−16.891, *P* < 0.001], Hepatitis B e antigen negativity [*OR*, 2.218; 95% CI, 4.335, *P* = 0.020], and additional N-glycosylation mutation [*OR*, 2.831; 95% CI, 1.157−6.929; *P* = 0.023] were independent risk factors for HCC in HBsAg/anti-HBs coexistent patients. Dynamical analysis showed that the additional N-glycosylation mutations existed 1-4 years prior to HCC occurrence in eight of 18 patients observed. In conclusion, the dditional N-glycosylation mutations together with HBsAg/anti-HBs coexistence might serve as a predictive indicator for HCC occurrence in chronic HBV-infected patients.

## INTRODUCTION

Hepatitis B virus (HBV) infection is a global pandemic disease that affects two billion people worldwide; up to 80% of hepatocellular carcinomas (HCC) are caused by HBV infection, and about 300,000 people die of HBV related HCC each year [1, 2]. Early diagnosis through intensive monitoring of populations at risk is critical for the management of HCC and comprehensive knowledge on risk factors associated with HCC occurrence is important. Both host and virus factors are involved in the occurrence of HBV-related HCC, while the knowledge of risk factors is still far from complete. Recently, high viral load, several patterns of mutations in HBV S, preS1 and preS2 regions were reported to be associated with the development of HCC [3, 4].

HBV envelope proteins is encoded by the preS/S gene, which includes the preS1, preS2 and S genes, encoding large protein (preS1 + preS2 + S), middle protein (preS2 + S), and small proteins or HBsAg (S). HBsAg includes glycosylated GP27 and non-glycosylated P24. The region of amino acids (aa) 99−169 in HBsAg is termed the major hydrophilic region (MHR), and it contains the major conformational epitope exposed on the external surface of the viral particle. Generally, the emergence of antibody to hepatitis B surface antigen (anti-HBs) leads to the elimination of infectious HBV. However, coexistence of detectable HBsAg and anti-HBs in serum samples has been reported in approximately 5% of patients chronically infected with HBV, [5, 6]. An investigation showed that the incidence of HCC in HBsAg/anti-HBs coexistent patients was higher than that in single HBsAg-positive patients [22.9% (11/48) *vs.* 7.9% (56/707), *P* = 0.002] [7]. In another investigation enrolled 1,042 patients chronically infected with HBV and followed the patients up for a median period of 4.3 years (range 1.0-22 years), showing that the probabilities of cumulative incidence of HCC at 5, 10, and 15 years were significantly higher in HBsAg/anti-HBs coexistent patients than in single HBsAg-positive patients at baseline (12.7%, 23.4%, 69.4% *vs.* 4.9%, 13%, 20.6%, respectively; *P* = 0.008) [8]. Thus, it was suggested that the coexistence of HBsAg and anti-HBs may be related to HCC.

MHR N-glycosylation mutations that introduced an additional Asn-X-Ser/Thr site (where X is any aa except proline) may influence viral characteristics [9, 10, 11]. One study, where 216 HBsAg/anti-HBs coexistent patients and 182 single HBsAg-positive patients were recruited, showed that the frequency of additional N-glycosylation mutations was significantly higher in HBsAg/anti-HBs coexistent patients than in single HBsAg-positive patients (21.75% *vs.* 0.55%, *P* < 0.01) [12]. Also, the study demonstrated that mutant HBsAg reacted weakly with anti-HBs and had better virion enveloping capacity compared to wild-type HBsAg, and that mutant strains had better virion enveloping and secretion capacity than wild-type strains in phenotypic analysis. Consistently, Salpini et al [13] verified that the additional N-glycosylation mutations studied, except for the S113N+T131N pattern, showed a drastic reduction in the quantification of strep-tagged HBsAg where the Architect and Bio-Rad assays (both targeting the HBsAg) were used. Thus, MHR additional N-glycosylation mutations were likely to be a contributor to the HBsAg/anti-HBs coexistent status.

Until now, however, there is a paucity of data directly demonstrating the association of the additional N-glycosylation mutations in the MHR of HBV with HCC occurrence in HBsAg/anti-HBs coexistent patients. Our study aimed to clarify this issue through cross-sectional and longitudinal analyses of the additional N-glycosylation mutations in a large number of patients.

## RESULTS

### Clinical features, HBV genotype, and additional N-glycosylation mutations of patients

Table 1 summarizes the clinical background, HBV genotypes, and the additional N-glycosylation mutation frequency in the three groups of patients. Of all 778 patients, 106 patients were infected with genotype B HBV and 664 patients were infected with genotype C HBV, 8 patients were infected with other genotype HBV. A slightly higher rate of genotype C was found in HCC patients than in CHB and ACLF patients. Compared to ACLF and CHB patients, HCC patients had a significantly higher frequency of the additional N-glycosylation mutations.

**Table 1:** Clinical features, HBV genotype, and additional N-glycosylation mutation in the 778 patients enrolled in the study

Item	HCC (n = 193)	CHB (n = 433)	ACLF (n = 152)	*P* Value
**Age (year)**	53.51 ± 10.94	39.80 ± 13.91	46.11 ± 11.5	< 0.001
**Sex (M/F)**	168/25	365/68	125/27	0. 532
**HBV genotype B/genotype C**	14/179	62/365	30/120	0.002
**Anti-HBs (+/-)**	76/117	205/228	7/145	< 0.001
**HBeAg (+/-)**	100/93	295/135	62/90	< 0.001
**HBeAb (+/-)**	109/84	188/242	65/87	0.007
**ALT (U/L)**	73.3 (27.5-81.5)	223.7 (40-262)	265.2 (57-349)	< 0.001
**TBIL (mmol/L)**	36.6 (15.2-21.25)	56.3 (11.5-52.2)	321.5 (206-306)	< 0.001
**HBV DNA (log_10_IU/ml)**	5.06 ± 1.82	5.51 ± 1.90	5.53 ± 1.60	0.013
**N-glycosylation mutations [n (%)]**	24 (12.37%)	19 (4.39%)	4 (2.63%)	< 0.001

HCC, hepatocellular carcinoma; CHB, chronic hepatitis B; ACLF, acute-on-chronic liver failure; ALT, alanine aminotransferase; TBIL, total bilirubin. Chi-squared analysis of variance (ANOVA), and nonparametric Wilcoxon signed-ranked test were used. *P* values less than 0.05 were considered to be statistically significant.

### Association of coexistence of HBsAg/anti-HBs or HCC with the additional N-glycosylation mutations

**Figure 1:**
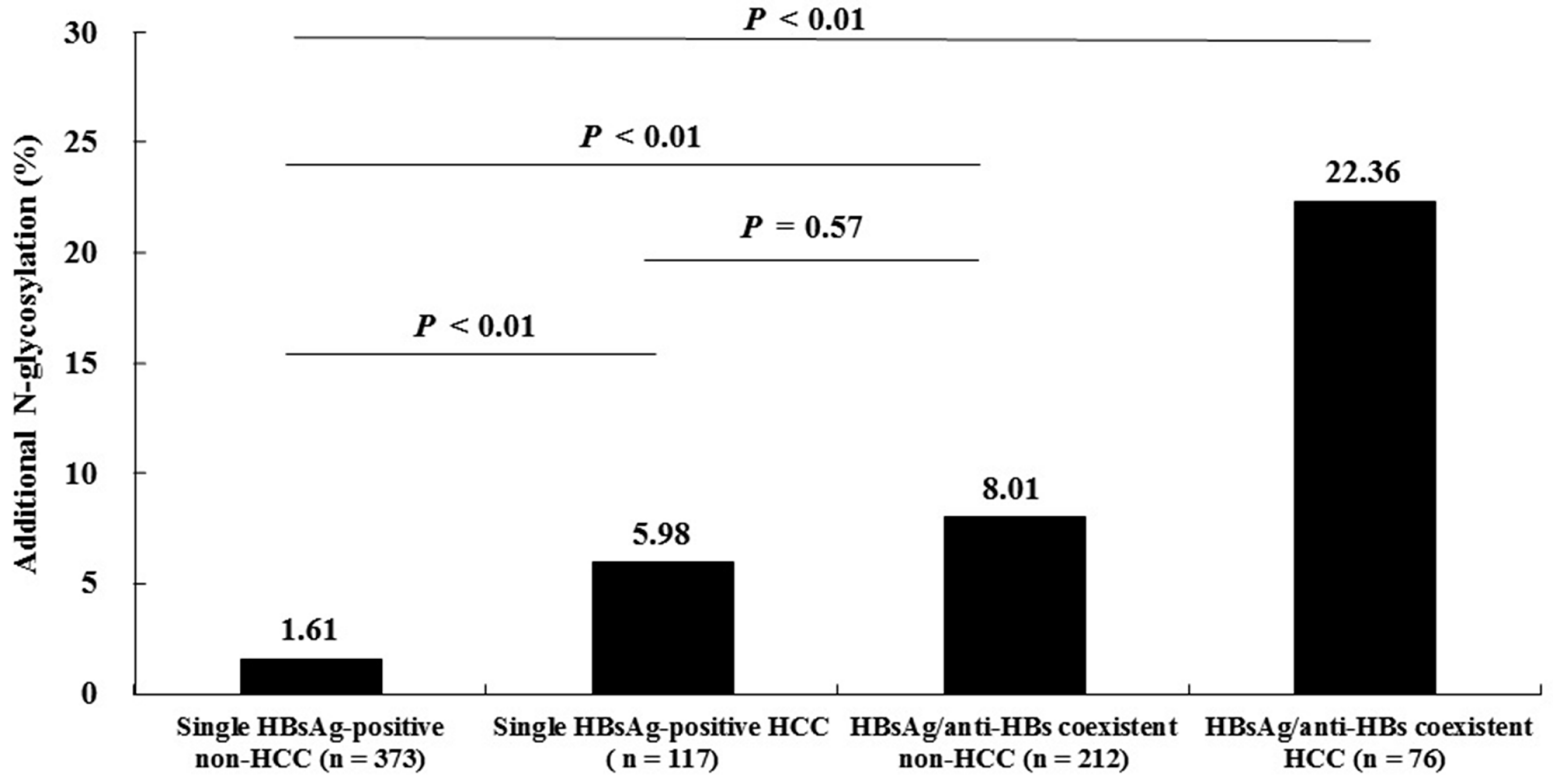
Comparison of the additional N-glycosylation mutation frequencies among four subsets of patients with different clinical presentations *P* values were calculated by chi-square test.

The presence or absence of the additional N-glycosylation mutations was determined in the 778 patients. These patients were classified into four groups as follows: Group I, single HBsAg-positive non-HCC (n = 373); Group II, single HBsAg-positive HCC (n = 117); Group III, HBsAg/anti-HBs coexistent non-HCC (n = 212); and Group IV, HBsAg/anti-HBs coexistent HCC (n = 76). As shown in Figure 1, the frequency of the additional N-glycosylation mutation increased in a stepwise manner from Group I to Group IV. The frequency differences were statistically significant except for the frequency difference between Group II and Group III. Specifically, group IV had a significantly higher frequency of the additional N-glycosylation mutations than that of either group II (22.36% *vs.* 5.98%, *P* < 0.01) or group III (22.36% *vs.* 8.01%, *P* < 0.01).

From another analytical perspective, the frequency of the additional N-glycosylation mutations were significantly higher in HCC patients (Groups II and IV) than in non-HCC patients (Groups I and III) (12.43% *vs.* 3.93%, *P* < 0.01), and higher in HBsAg/anti-HBs coexistent patients (Groups III and IV) than in single HBsAg-positive patients (Groups and II) (11.81% *vs.* 2.65%, *P* < 0.01).

To exclude the potential influence on the results brought by age differences among patient groups, patients aged from 30 to 46 and 47 to 66 years were analyzed as independent two subsets. Patients in different illness categories had a similar age within each subset; the frequencies of the additional N-glycosylation mutations remained significantly higher in HCC patients than that in ACLF and CHB patients (Supplementary Table 1). In addition, no significant difference in age between HCC patients with and without the N-glycosylation mutations was observed (54.46 ± 11.60 *vs.* 53.12 ± 10.69, *P* = 0.322); and no significant difference in age between the HCC patients with coexistent HBsAg/anti-HBs and the HCC patients with single HBsAg was observed (53.03 ± 10.50 *vs.* 53.66 ± 11.03, *P* = 0.692).

### Comparison of clinical and virological characteristics between HBsAg/anti-HBs coexistent HCC and non-HCC patients

Table 2 summarizes the clinical background, HBV genotypes, and the additional N-glycosylation mutation frequency in 288 HBsAg/anti-HBs coexistent patients. The overall frequency of the MHR additional N-glycosylation mutations in the 288 patients with coexistent HBsAg/anti-HBs was 11.81% (34/288). Specifically, the frequency of the additional N-glycosylation mutations were significantly higher in HCC patients than in non-HCC patients [22.38% (17/76) *vs.* 8.01% (17/212); *P* < 0.01].

**Table 2:** Clinical features, HBV genotype, and additional N-glycosylation mutation in the 288 patients with coexistence of HBsAg/anti-HBs enrolled in the study

Item	HCC ( n = 76)	Non-HCC ( n = 212)	*P* value
Age	53.03 ± 10.50	43.99 ± 9.89	< 0.001
Male [n (%)]	60 (78.94%)	174 (82.08%)	0.549
**HBV DNA (log_10_ IU/L)**	4.82 ± 1.61	5.32 ± 1.69	0.041
Anti-HBs (IU/L)	52.04 (26-140)	42.97 (22-100)	0.085
TBIL (μmol/L)	19.25 (12-27)	16.75 (10-32)	0.146
ALT (U/L)	31 (26-39)	49.5 (22-44)	< 0.001
ALB (g/L)	32.11 ± 6.25	37.22 ± 8.12	< 0.001
AFP (ng/mL)	28.15 (11-452)	11 (6-31)	< 0.001
CHE (U/L)	6406 ± 1663.68	5368 ± 2299.46	0.001
HBV genotype C	75 (98.68%)	198 (93.40%)	0.075
N-glycosylation mutations [n (%)]	17 (22.38%)	17 (8.0%)	0.001

TBIL, total bilirubin; ALT, alanine aminotransferase; ALB, albumin; AFP, alpha-fetoprotein; CHE, cholinesterase. Chi-squared, Student *t* test analysis, and nonparametric Wilcoxon signed-ranked test were used. A *P* value less than 0.05 was considered to be statistically significant.

Twenty kinds of additional N-glycosylation mutations/mutation patterns were detected. Among them, five mutation patterns have not been reported previously, as follows: (1) 112-113 “KNA” insertion→114-116NAS; (2) 114-115 “NTSTT” insertion→115-117NTS; (3) sT113N+114-116 “STT” deletion→113-115NST & sT131N+M133T→131-133NST; (4) 115-116 “INGTST” insertion→117-119NGT; and (5) sT116N→116-118NST & sT131N+M133T→131-133NST (Table 3). The dominant mutation pattern was sT131N+M133T→131-133NST, which ocupied 58.82% (20/34).

**Table 3:** Clinical features and additional N-glycosylation mutational patterns in 34 HBsAg/anti-HBs coexistent patients

Sample ID	Sex M/F	Age (yr)	HBsAg IU/ml	Anti-HBs IU/L	HBeAg COI	HBV DNA log_10_ IU/ml	Genotype	N-glycosylation site	Clinical diagnosis
S6178	M	31	6752	39.84	1.53	2.39	C	sQ129N→129-131NGT	HCC
S1454	M	44	1255	35.3	0.086	3.09	C	sG130N+T131N→130-132NNS	HCC
S8812	M	46	150.9	24.43	619.4	6.46	C	sT131N+M133T→131-133NST	HCC
S11002	M	46	5.62	14.61	8.34	4.51	**C**	**112-113 “KNA” insertion→114-116NAS**	HCC
S14383	M	48	23.68	56.21	0.244	4.54	C	sT131N+M133T→131-133NST	HCC
S14087	M	49	66.83	173.6	0.598	4.06	**C**	sQ129N→129-131NGT	HCC
S3771	M	50	6314	117.2	1.56	4.77	C	sT131N+M133T→131-133NST	HCC
S13977	M	51	22.9	142	17.35	4.09	C	sT131N+M133T→131-133NST	HCC
S12744	M	54	360.9	10.65	0.11	3.3	C	sT116N→116-118NST	HCC
S6658	M	57	24.42	467.9	2.07	3.91	**C**	**114-115 “NTSTT” insertion→115**-**117NTS**	HCC
S6765	M	58	91.86	56.33	0.076	<2	C	sT131N+M133T→131-133NST	HCC
S14469	M	61	584.4	21.18	98.3	6.32	C	sT131N+M133T→131-133NST	HCC
S1504	M	66	381.7	117.1	15.89	2.84	C	sT131N+M133T→131-133NST	HCC
S4181	F	67	2913	115.9	0.115	7.25	C	sG130N→130-132NTS	HCC
S12077	F	79	2.13	155.2	0.083	3.2	C	**sT113N+114-116 “STT” deletion→113**-**115NST & sT131N+ M133T→131-133NST**	HCC
S4	M	82	3295	2.15	0.084	<2	C	sG130N+T131I→130-132NIS	HCC
S80	M	49	6561	15.29	0.123	<2		**115-116 “INGTST” insertion→117-119NGT**	HCC
S6422	F	51	3992	172.2	453.7	7.19	C	sG130N→130–132NTS	ACLF
S2321	F	11	3588	683.3	159.6	7.76	C	sT131N+M133T→131-133NST	CHB
S6672	M	38	2680	202.7	0.702	6.64	C	sT131N+M133T→131-133NST	CHB
S1912	M	39	5329	24.78	19.11	4.13	C	sT131N+M133T→131-133NST	CHB
S3748	M	41	6117	42.97	178.8	6.64	C	sT131N+M133T→131-133NST	CHB
S13246	M	48	29.83	35.45	391.6	7.18	C	sT131N+M133T→131-133NST	CHB
S12931	M	53	1.848	61.97	67.03	7.49	C	**sT116N→116-118NST & sT131N+ M133T→131-133NST**	CHB
S2521	M	54	1285	122.2	1463	8.11	C	sT131N+M133T→131-133NST	CHB
S2358	M	36	4366	18.7	0.17	3.33	C	sT131N+M133T→131-133NST	LC
S4445	F	37	107.1	18.01	86.79	6.89	B	sT131N+M133T→131-133NST	LC
S11103	M	38	1.98	28.25	44.13	2.84	C	sT113N→113-115 NST	LC
S823	M	45	1.53	26.44	11.38	4.01	C	114-115“TTN” insertion→NST	LC
S8893	M	49	237.1	139.3	0.111	5.5	C	sT131N+M133T→131**-**133NST	LC
S7259	M	52	246	34.2	1269	7.95	C	sT131N+M133T→131-133NST	LC
S15938	M	56	1216	33.01	0.08	4.8	C	sT131N+M133T→131-133NST	LC
S7883	M	57	88.89	71.19	816.7	6.97	C	sT131N+M133T→131-133NST	LC
S9862	M	61	57.38	44	0.104	3.22	C	sG130N→130-132NTS	LC

Five unreported mutations/mutation patterns are expressed in bold. HCC, hepatocellular carcinoma; ACLF, acute-on-chronic liver failure; CHB, chronic hepatitis B; LC, liver cirrhosis.

In the HCC patients with coexistence of HBsAg/anti-HBs, there were eight additional N-glycosylation mutation patterns, including aa point mutations at s116, s129, s130 and s131, a 3-aa (KNA) insertion between s112 and 113, a 5-aa (NTSTT) insertion between s114 and 115, a 3-aa (STT) deletion between s114 and 115, and a 6-aa (INGTST) insertion between s115 and 116. In the non-HCC patients with coexistence of HBsAg/anti-HBs, There were five additional N-glycosylation mutation patterns, including aa point mutations at s113, s116, s130, and s131, and a 3-aa (TTN) insertion between s114 and 115. These point mutations, insertions, or deletions introduced an additional “N-X-T/S” motif.

**Figure 2:**
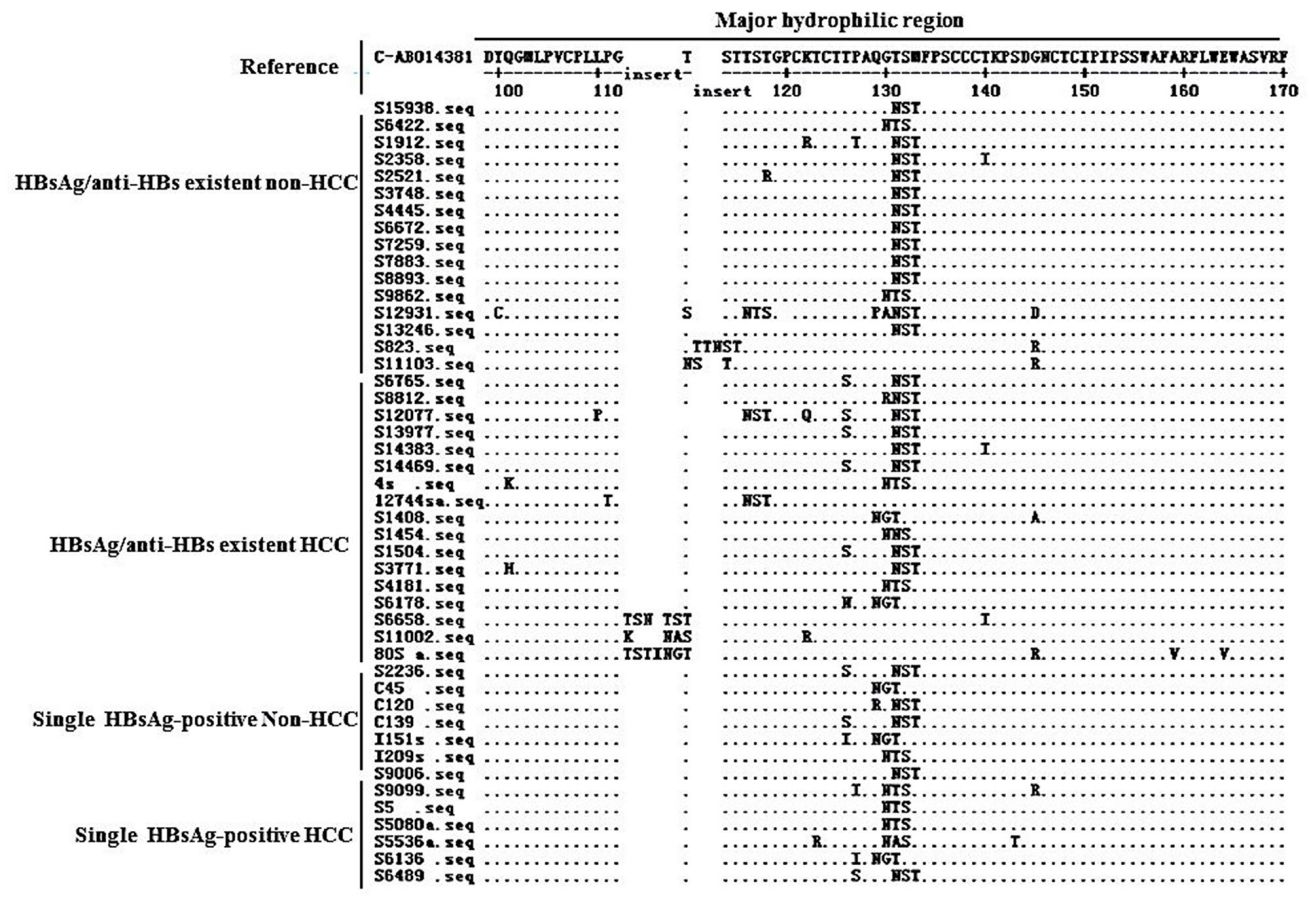
Patterns of the 34 patients with additional N-glycosylation site within the MHR of HBsAg in patients with coexistence of HBsAg/anti-HBs

Alignment of MHR aa sequences showed that HCC patients had a relatively higher frequency (6/17) of sT126N/S concomitant with an additional N-glycosylation mutation, compared to non-HCC patients (0/17) (Figure 2).

### Confirmation of genetic N-glycosylation mutations at translational level

Given that the conversation of classical N-glycosylation genetic mutations to amino acid substitutions have been proved in previous reports, we focused on the confirmation of the N-glycosylation genetic mutations that we firstly reported. Reverse site-directed mutagenesis was conducted for one novel mutant with dual N-glycosylation mutations together with a concomitant mutant that harbored a single N-glycosylation mutation in the patient’s samples. Before PNGase F treatment, there were two bands visible in the wild-type protein that represented the inherent N146 glycosylated and non-glycosylated bands. In comparison, the mutants with one and two additional N-glycosylation mutation(s) had three and four bands, respectively. After PNGase F treatment, both the wild-type and mutant sequences had a single non-glycosylated band that was consistent in mass to HBsAg without glycosylation (Supplementary Figure 1A and 1B).

### Multivariate analysis of the risk factors for HCC in HBsAg/anti-HBs coexistent patients

In univariate analysis, age > 40 years, liver cirrhosis,AFP > 10 ng/mL, HBeAg negativity, and the additional N-glycosylation mutations were significantly associated with HCC in HBsAg/anti-HBs coexistent patients. In multivariate logistic regression analyses, age > 40 years, AFP > 10 ng/mL, liver cirrhosis, HBeAg negativity, and the additional N-glycosylation mutations [OR, 2.831; 95% CI, 1.157−6.929; *P* = 0.023] were independent risk factors for the occurrence of HCC in HBsAg/anti-HBs coexistent patients (Table 4).

**Table 4:** Univariate and multivariate analyses of risk factors for predicting HCC with coexistence of HBsAg/anti-HBs development.

Factors	HCC (n = 76)	Non-HCC (n = 212)	Univariate	Multivariate	
			*P* value	Odds ratio	*P* value
Male [n (%)]	60 (78.94%)	173 (81.60%)	0.549	0.548 (0.236**-**1.277)	0.164
Age >40	69 (90.79%)	132 (70.97%)	< 0.001	3.005 (1.177**-**7.674)	0.021
AFP >10 ng/mL	59 (77.63%)	86 (40.57%)	< 0.001	4.718 (2.406**-**9. 251)	< 0.001
Liver cirrhosis	68 (89.45%)	115 (54.24%)	< 0.001	6.844 (2.773**-**16.891)	< 0.001
HBV genotype C [n (%)]	75 (98.68%)	198 (93.40%)	0.075	7.960 (0.847**-**74.841)	0.070
HBV DNA (log_10_ IU/mL)					
1 (2.23-3.99)	26 (34.21%)	45 (21.22%)	0.068	1.320 (0.392**-**4.440)	0.654
2 (4-5.99)	22 (28.94%)	69 (32.55%)	0.607	1.008 (0.304**-**3.334)	0.990
3 ( ≥6)	20 (26.31%)	75 (35.38%)	0.168	0.773 (0.220**-**2.438)	0.612
HBeAg negative [n (%)]	33 (43.42%)	50 (23.85%)	< 0.001	2.218 (1.134**-**4.335)	0.020
N-glycosylation mutation [n (%)]	17 (22.37%)	17 (8.02%)	< 0.001	2.831 (1.157**-**6.929)	0.023

95% CI, confidence interval; ALT, alanine aminotransferase; AFP, alpha-fetoprotein; HCC, hepatocellular carcinoma.

### Longitudinal observation of the additional N-glycosylation mutation with the occurrence of HCC in HBsAg/anti-HBs coexistent patients

Among HCC patients enrolled in the study, sequential serum samples collected 2-4 years before HCC diagnosis were available from 96 patients. These included 78 patients with persistent single positivity of HBsAg (defined as the control group) and 18 patients with coexistent HBsAg/anti-HBs at one sampling time-point at least (defined as the case group). The frequency of the additional N-glycosylation mutations in the case group was significantly higher than that in the control group [44.44% (8/18) *vs.* 14.10% (11/78), *P* < 0.01]. Regarding the eight patients with coexistent HBsAg/anti-HBs samples, HBsAg/anti-HBs coexistence was detected at three time-points for 6/8 patients, at three time-points for 1/8 patient and at four time-points for 1/8 patients. Of these eight patients, some patients exhibited a sequential process of additional N-glycosylation mutation development, HBsAg/anti-HBs presentation, and HCC occurrence. The additional N-glycosylation mutation and coexistent HBsAg/anti-HBs before and at HCC diagnosis were consistently found in four patients (P2, P3, P5, P6); the additional N-glycosylation mutations were consistently found in three patients (P1, P7, P8), but anti-HBs became negative at HCC diagnosis. The remaining patient (P4) was negative for both additional N-glycosylation mutation and anti-HBs at HCC diagnosis.

### Influence of the additional N-glycosylation mutations on HBV replication capacity

HBV replication capacity was reflected by intracellular HBV replicative intermediate level. The examination was performed for the 13 mutants (M1−M13) with additional N-glycosylation mutations in MHR of S gene. Compared to the wild-type strain, no significant difference was observed in intracellular replicative intermediate levels between each mutant and the wild-type (Supplementary Figure 2). The difference between each mutant and the wild-type was not statistically significant (*P* all > 0.05).

### Influence of the additional N-glycosylation mutations on cell proliferation and migration

Five representative additional N-glycosylation mutants were examined. The mutation patterns were 115-116“NGTST” insertion→117-119NGT for M1, 112-113“KNA” insertion→114-116NAS for M2, 114-115“NTSTT” insertion→115-117NTS for M3, sT116N→116-118NST & sT131N+M133T→131-133NST for M4, and sT131N+M133T→131-133NST for M5. Among them, M1-M4 were firstly identified by our team. The cell proliferation of the HepG2 cells transfected with mutant recombinants were all comparable compared to the HepG2 cells transfected with wild-type recombinant (Supplementary Figure 3). Consistently, the cell migration of the HepG2 cells transfected with mutant recombinants were all comparable compared to the HepG2 cells transfected with wild-type recombinant (Supplementary Figure 4).

## DISCUSSION

The identification of risk factors for HCC occurrence is currently a topic of intense research. A few studies have reported that HCC is more frequently observed in HBsAg/anti-HBs coexistent patients than in single HBsAg-positive patients; however, the underlying mechanisms are not fully understood [7, 8]. In this study, we found that the frequency of the additional N-glycosylation mutations in the MHR of HBV S gene was significantly higher in HBsAg/anti-HBs coexistent patients than in single HBsAg-positive patients, suggesting that the additional N-glycosylation mutation may play an important role in the HBsAg/anti-HBs coexistent status.

Until now, data are very limited regarding the additional N-glycosylation mutation in HCC patients with coexistent of HBsAg/anti-HBs. One previous study showed that the frequency of the additional N-glycosylation mutations were significantly higher in HBsAg/anti-HBs coexistent patients with HCC (8/15, 53%) than in HBsAg/anti-HBs coexistent patients with CHB (28/129, 29%), immune tolerant HBV infection (8/50, 16%), or HBV-related liver cirrhosis (2/19, 11%) [12]. However, this study did not report a significant difference in the frequency of the additional N-glycosylation mutation among these illness categories in single positive HBsAg patients. By contrast, we found that the frequency of the additional N-glycosylation mutation in the MHR was significantly higher in HCC patients than in non-HCC patients, not only in those with coexistent HBsAg/anti-HBs (HCC 22.36% *vs.* non-HCC 8.01%, *P* < 0.01), but also in those with single-positive HBsAg (HCC 5.98% *vs.* non-HCC 1.61%, *P* < 0.01) (Figure 1). The reason for the difference in these findings could be the greater number of samples included in our study. Thus, our results showed that the HBsAg/anti-HBs coexistent HCC patient group had a higher frequency of the additional N-glycosylation mutation than the other three patient groups (HBsAg/anti-HBs coexistent non-HCC patients, single HBsAg-positive HCC patients, and single HBsAg-positive non-HCC patients) (Figure 1). In addition, the frequency of the additional N-glycosylation mutation in the MHR was significantly increased in HBsAg/anti-HBs coexistent non-HCC patients and in single HBsAg-positive HCC patients, compared to in single HBsAg-positive non-HCC patients; while the mutation frequency was similar between the first two groups of patients. These results have three implications: (1) they provide stronger evidence for the positive association of the additional N-glycosylation mutations in the MHR with HCC occurrence; (2) they suggest that the association of the additional N-glycosylation mutation in the MHR is not limited to its contribution to HBsAg/anti-HBs status; and (3) the additional N-glycosylation mutation in the MHR and HBsAg/anti-HBs coexistence have a compound effect on the prediction of HCC occurrence.

Therefore, we proposed that the additional N-glycosylation mutation in the MHR might have a direct impact on HCC occurrence, although the mechanism remains unclear. Recently, it was proposed that an HBV preS mutant increased HCC occurrence by activating both endoplasmic reticulum (ER) stress-dependent and ER stress-independent signals [14]. Liu et al [15] reported that the N-glycosylation modification of LHBs affects ER stress or expression of cycling, cell cycle, and proliferation, which was associated with HBV-related HCC occurrence. Although our preliminary experiments in current study did not show significant influence of the additional N-glycosylation mutations on cell proliferation and migration, further study remains needed to clarify whether the additional N-glycosylation mutations has direct tumorigenicity potential. On the other hand, the additional N-glycosylation mutations are suggested to be related to immune escape because hyperglycosylation can shield surface exposed epitopes in the MHR from neutralizing anti-HBs antibodies and might elicit low-affinity or non-neutralizing antibodies [11]. Previous studies including ours really verified that several addition N-glycosylation mutations in the MHR significantly attenuated HBsAg antigenicity which may lead to the incomplete binding of anti-HBs to HBsAg, negatively influencing HBV clearance, and therefore beneficial to viral immune escape [10, 12, 16].

In our study, multivariate analysis was performed to verify that the additional N-glycosylation mutation was an independent risk factor for HCC. Besides, we performed age stratification analysis and comparison of ALT levels between HCC and non-HCC patients. The results showed that both age and ALT levels had no significant influence on the N-glycosylation mutation occurrence (Supplementary Tables 1 and 2). Adding a subgroup of liver cirrhosis patients will consolidate the conclusion. To circumvent this limitation, we performed analysis by comparing the N-glycosylation mutation frequencies between the acute-on-chronic liver failure (ACLF) patients with liver cirrhosis and those without liver cirrhosis. In ACLF group, 63.16% (96/152) patients were with liver cirrhosis and 36.84% (56/152) patients were without liver cirrhosis. There was no significant difference in the frequency of the N-glycosylation mutation between ACLF patients with and without liver cirrhosis [2.08% (2/96) *vs.* 1.31% (2/152), *P* = 0.640]. In addition, no significant difference in the frequency of the N-glycosylation mutation was observed between HCC patients with liver cirrhosis and those without liver cirrhosis [12.29% (22/179) *vs.* 14.29% (2/14), *P* = 0.828].

Whether nucleot(s)ide analog (NA) antiviral treatment had potential influence on the N-glycosylation mutation occurrence was also analyzed. In our study, 73.01% (568/778) of the enrolled patients had received NA treatment at sampling, and NA therapies received by HCC and non-HCC patients were comparable in general. There was no significant difference in NA exposure between the patients positive for the N-glycosylation mutations and those negative for the N-glycosylation mutations [70.21% (33/47) *vs.* 73.19% (535/731), *P* = 0.656)]. In addition, there was no significant difference in detection rate of primary NA-resistant mutations between the samples positive for the N-glycosylation mutations and those negative for the N-glycosylation mutations [21.28% (10/47) *vs.* 18.88% (138/731), *P* = 0.685)]. The results indicated that NA antiviral treatment which targets HBV reverse transcriptase region had no significant association with the occurrence of the MHR N-glycosylation mutations in HBV S region.

In this study, we found that five of additional N-glycosylation mutation/mutation patterns have not been reported in previous publications. The characteristics of the five mutation/mutation patterns were distinct from classic ones and could be classified into two types: (1) with a 3-5 aa insertion forming an additional N-glycosylation mutation site; and (2) with double additional N-glycosylation mutation sites in the MHC (Table 4). Interestingly, the first type of mutation pattern was observed in HCC patients; while the second type of mutation pattern was observed in a CHB patient. The five additional N-glycosylation mutation/mutation patterns were not detected in single HBsAg-patients with either HCC or non-HCC (Figure 2).

**Figure 3:**
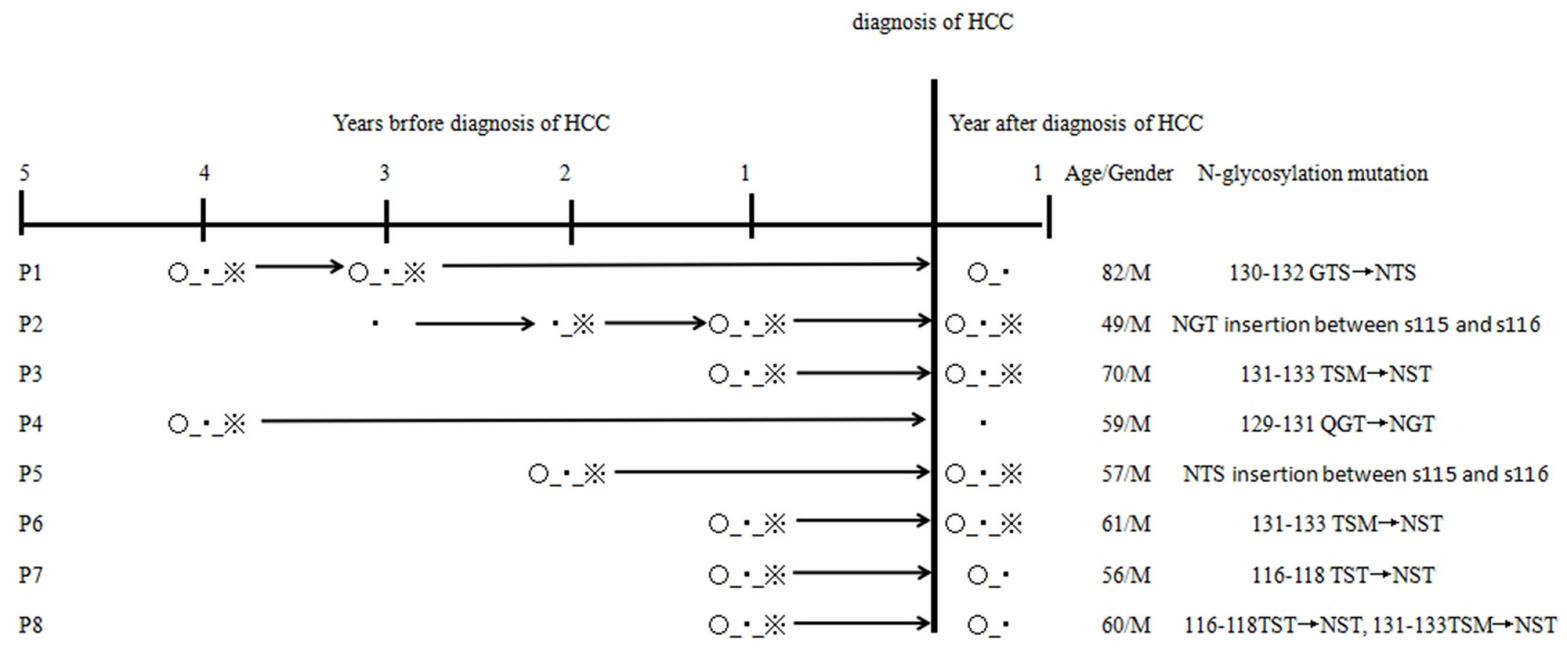
Longitudinal observation of additional N-glycosylation mutation in 8 HCC patients with coexistence of HBsAg/anti-HBs ○, the N-glycosylation mutation positive; ▪, HBsAg positive; ※, anti-HBs positive.

We were able to retrospectively collect serial serum samples from 96 HCC patients, including samples from 18 HBsAg/anti-HBs coexistent patients. Among them, additional N-glycosylation mutations in the MHC were detected 1-4 years prior to HCC occurrence (Figure 3). Similarly, longitudinal observation of HBV preS deletions in HCC occurrence was reported in previous studies [7, 17, 18], reinforcing the evidence for the involvement of HBV mutation in HCC occurrence. Further study is required for clarifying whether these viral mutations are the driving factors or just indicators.

In conclusion, additional N-glycosylation mutation in the MHR of HBV S gene is more frequently detected in HBsAg/anti-HBs coexistent HCC patients compared to HBsAg/anti-HBs coexistent non-HCC patients or single HBsAg-positive HCC patients. The additional N-glycosylation mutations together with HBsAg/anti-HBs coexistence might serve as a predictive indicator for HCC occurrence in chronic HBV-infected patients. This study provides new insights into HBV mutations in HCC and it will be helpful in the management of the disease.

## MATERIALS AND METHODS

### Patients and serum samples

Sera of 778 patients who were admitted to, or visited, the Beijing 302 Hospital from January 2009 to December 2015 were collected and successfully amplified for the HBV S and a 684-bp-long S/Pol (nt 155–838) gene regions. The patient cohort comprised 194 HCC, 433 CHB, and 146 ACLF patients. The patients came from various areas of China, but mainly from the north of the country. The diagnostic criteria were based on the Guideline of Prevention and Treatment for Chronic Hepatitis B [19] and Diagnostic and Treatment Guidelines for Liver Failure [20]. Specifically, CHB was defined as a history of HBsAg for more than 6 months, persistent elevation in serum ALT levels, and a history of chronic hepatitis based on a histopathological diagnosis and/or compatible laboratory data and ultrasonographic findings. Liver cirrhosis and HCC were diagnosed by imaging tests such as CT scan, MRI and ultrasound, liver biopsy, and/or AFP serology. ACLF patients met the following criteria as described in our previous study [21]: recent development of increasing jaundice (TBIL > 171.0 μmol/L or rapid increase to > 17.1 μmol/L/day) and decreasing PTA (< 40%), with a recent development of complications, such as hepatic encephalopathy (≥ grade 2), or an abrupt and obvious increase of ascites or spontaneous bacterial peritonitis or hepatorenal syndrome. For all patients, there was no evidence for concomitant hepatitis C/D virus (HCV/HDV) or human immunodeficiency virus (HIV) infection or autoimmune liver disease. At the same time, we randomly selected two CHB patients, from HBsAg-positive patients who were alive and have not been diagnosed with HCC throughout the follow-up period. A total of 96 controls were enrolled. These patients were from the database of Bejing 302 Hospital and all provided their informed consent before enrollment in the Database of Beijing 302 Hospital. The study was approved by the Ethics Committee of Beijing 302 Hospital.

### Serological markers and quantitation of HBV DNA

All enrolled patients were hospitalized at least for one time in Beijing 302 Hospital. Blood samples were obtained at initial administration of the patients to Beijing 302 Hospital and either immediate use or stored in Serum Bank of the Database of Beijing 302 Hospital until use. The detection of biochemical and serological parameters and HBV DNA level were routinely performed in the Central Clinical Laboratory of the Beijing 302 Hospital.

### Sequence amplification and analysis

Viral DNA was extracted and subjected to a nested PCR as described elsewhere [22]. The primers were 5′-TCGCAGAAGATCTCAATCTCG-3′ [sense, nucleotide (nt) 2416−2436) and 5′- AGGTGAAGCGAAGTGCAC AC-3′ (antisense, nt 2403−2422) for the first-round PCR and 5′- TTCCGCAGTATGGATCGGCAG -3′ (sense, nt 1258−1278) and 5′-CATAAGGTGGGAAACTTTAC -3′ (antisense, nt 2466−2485) for the second-round PCR. The first-round PCR consisted of pre-denaturation at 94°C for 5 min, followed by 30 cycles of 94°C for 40 s, 57°C for 1 min 12 s, and 72°C for 2 min 24 s, and extension at 72 for 10 min. The second-round PCR consisted of 94°Cfor 5 min, followed by 30 cycles of 94°C for 40 s, 57°C for 1 min, and 72°C for 2 min, and extension at 72°C for 10 min. Sequencing was performed using an ABI3730xl DNA Analyzers (Applied Biosystems, Foster City, CA). Analysis and assembly of sequencing data were performed with the Vector NTI Suite software package (Informax, Frederick, MD). Mutations for both additional N-glycosylation mutations in MHR of S gene and primary drug-resistant mutations in transcriptase reverse region of polymerase gene were analyzed. The definition and analysis of primary drug-resistant mutations have been described in our previous study [23].

### Typing of HBV genotypes

The genotypes were determined based on complete genomic sequence or on analysis of the 1225-bp-long S/Pol-gene sequence, as we previously described [24, 25]. Briefly, HBV genotyping was performed by direct PCR sequencing followed by molecular evolutionary analysis of the viral sequences using MEGA 4 software. Standard reference sequences were acquired from the online Hepatitis Virus Database (http://www.ncbi.nlm.nih.gov/projects/genotyping/formpage.cgi).

### Recombinant plasmid construction

Two different types of recombinant plasmids for mutant or wild-type sequences were used in this study. The first was on the basis of pTriEx-mod-1.1 HBV vector (a kind gift from Professor Zoulim, University Lyon, France), which was used for the measurement of HBV replicative intermediates. HBV preS/S-gene fragments were inserted into the *Bst*EII and *Sph*I restriction sites of the vector. The second was on the basis of pcDNA3.1(-)/myc-His A vector (Invitrogen, Carlsbad, CA, USA). The fusion protein produced from this vector has a C-terminal polyhistidine (His) tag which can be detected using anti-His-tag antibody and used for the confirmation of N-glycosylation mutations at translational level, as well as for the assessment of the mutations on cell proliferation and migration potentials. S-gene fragments were cloned into the *Eco*RI and *Kpn*I restriction sites of this vector. All restriction enzymes were purchased from New England BioLabs (NEB, Ipswich, MA, USA).

### Site-directed mutagenesis

One representative mutant with dual N-glycosylation mutations (M4: sT116N→116-118NST & sT131N+M133→131-133NST) and one representative mutant with single N-glycosylation mutation (M5: sG130N+T131S→130-132NSS) were selected for the removal of N-glycosylation mutation(s) using reverse site-directed mutagenesis. The method was described in our previous studies with minor modifications [26]. In brief, a QuikChange Lightning site-directed mutagenesis kit (Stratagene, La Jolla, CA, USA) was employed according to manufacturer’s instructions to eliminate mutation at specific site. The mutagenic primers annealing to the same sequence on opposite strands of the plasmids were designed individually, and PCR was performed to synthesize the mutation-eliminated strand. For M4 that harbors dual N-glycosylation mutations, the elimination was performed in stepwise, i.e., to generate M4’a (sT116N→116-118NST) and M4’b (sT131N+M133) respectively first, followed by generating M4’c (without N-glycosylation) using M4’a as template. *Dpn*I restriction enzyme was added to digest parental methylated and hemimethylated DNA. The reversely mutated sequence was transformed into competent cells XL-10 for nick repair. The laboratory-modified wild-type gene fragment was linked with pcDNA3.1(-)/myc-His A vector as mentioned above.

### Confirmation of additional N-glycosylation mutations

Each additional N-glycosylation at aa level will have an approximate 3-kDa increase in molecular weight. Whether genetic N-glycosylation truly converted to aa N-glycosylation was confirmed by de-glycosylation using PNGase F treatment. PNGase F (P0704, NEB) was used to remove N-linked glycans from recombinant HBsAg that had N-linked glycosylation sites, according to the manufacturer’s instructions. Cells were lysed with RIPA buffer by adding 1 mmol/L PMSF. Afterwards, the samples were subjected onto 12% SDS-PAGE gel before transferal to a PVDF membrane. Glycosylation and de-glycosylation HBsAg were paralleled detected by Western blotting using a mouse monoclonal anti-His-tag antibody as the primary and then visualized using HRP-labeled goat anti-mouse IgG (HS201-01, TransGen Biotech) secondary antibody in combination with a SuperSignal West Pico Chemiluminescent Substrate or West Femto Maximum Sensitivity Substrate. A marker (MagicMark XP Western Protein Standard, Invitrogen) was used to estimate molecular weight.

### Quantitation of intracellular replicative intermediates

The experiments were performed as we described previously [16, 27]. In brief, HepG2 cells were seeded onto six-well plates. The recombinant pTriEx-mod-1.1HBV vectors were transfected into HepG2 cells mediated by X-treme GENE HD transfection reagent (Roche,Mannheim, Germany). As a control, the β-galactosidase reporter plasmid was co-transfected to normalize transfection efficiency. The transfection efficiencies within an experiment and across independent experiments were comparable. After 4-day cultivation, cells and culture supernatant were harvested for quantification of intracellular replicative intermediates using a real-time quantitative PCR kit (Fosun Pharmaceutical Co., Ltd., Shanghai). The experiments were performed at least three times independently.

### Cell proliferation assay

The experiment was conducted as described elsewhere with minor modifications [28]. In brief, cell Counting Kit (CCK)-8 (TransGen Biotech, Beijing, China) was used to determine cell proliferation according to the manufacturer's instructions. HepG2 cells were transfected with either mutant or wild-type S-gene recombinant pcDNA3.1(-)/myc-His A plasmids. The cells were seeded onto 96-well plates at 2000 cells/well after 12 h of the transfection,. At 12, 24, 36, 48 and 60 h, the culture medium in each well was replaced with 200 ml fresh medium mixed with 10 ml CCK-8 solution. The absorbance of each well was measured using a microplate reader (BioTek, USA) at 450 nm. The experiment was independently performed for three times.

### Wound healing assay

Wound healing assay was performed to evaluate the cell migratory capacity. In brief, HepG2 cells that had been transfected with either mutant or wild-type S-gene recombinant pcDNA3.1(-)/myc-His A plasmid were cultured to full confluence. Wounds of approximately 1 mm width were created with a plastic scriber, and cells were washed and above plasmid vectors were transfected. Briefly, 48 h after the transfection of HepG2 cells, cells were fixed and observed under a microscope as described elsewhere [29]. At least six microscopy images were examined for each sample and the experiments were performed twice.

### Statistical analysis

Continuous variables were expressed as mean ± standard deviation (SD) or median. Differences in continuous data were evaluated using Student’s *t* test, analysis of variance (ANOVA), or nonparametric Wilcoxon signed-ranked test, where appropriate, and categorical data were analyzed using the chi-square test and Fisher’s exact test. Multivariate analysis with logistic regression was used to determine independent factors. Statistical analysis was carried out in SPSS version 18.0 software (SPSS, Chicago, IL). *P* values of < 0.05 and < 0.01 (two-sided) were considered to be statistically significant and very significant, respectively.

## SUPPLEMENTARY MATERIALS FIGURES AND TABLES



## Abbreviations

HCC: hepatocellular carcinoma
CHB: chronic hepatitis B
ACLF: acute-on-chronic liver failure
ALT: alanine aminotransferase
TBIL: total bilirubin
ALB: albumin
PTA: prothrombin activity
AFP: alpha-fetoprotein
OR: odds ratio
95% CI: confidence interval

## References

[1] Liang X, Bi S, Yang W, Wang L, Cui G, Cui F, Zhang Y, Liu J, Gong X, Chen Y, Wang F, Zheng H, Wang F (2013). Reprint of: epidemiological serosurvey of hepatitis B in China-declining HBV prevalence due to hepatitis B vaccination. Vaccine.

[2] Lafaro KJ, Demirjian AN, Pawlik TM (2015). Epidemiology of hepatocellular carcinoma. Surg Oncol Clin N Am.

[3] Coppola N, Onorato L, Iodice V, Starace M, Minichini C, Farella N, Liorre G, Filippini P, Sagnelli E, de Stefano G (2016). Occult HBV infection in HCC and cirrhotic tissue of HBsAb-negative patients: a virological and clinical study. Oncotarget.

[4] Chen X, Wu F, Liu Y, Lou J, Zhu B, Zou L, Chen W, Gong J, Wang Y5, Zhong R (2016). The contribution of serum hepatitis B virus load in the carcinogenesis and prognosis of hepatocellular carcinoma: evidence from two meta-analyses. Oncotarget.

[5] Colson P, Borentain P, Motte A, Henry M, Moal V, Botta-Fridlund D, Tamalet C, Gerolami R (2007). Clinical and virological significance of the co-existence of HBsAg/anti-HBs antibodies in hepatitis B chronic carriers. Virology.

[6] Pondé RA (2011). The underlying mechanisms for the “simultaneous HBsAg/anti-HBs serological profile”. Eur J Clin Microbiol Infect Dis.

[7] Jang JS, Kim HS, Kim HJ, Shin WG, Kim KH, Lee JH, Kim HY, Kim DJ, Lee MS, Park CK, Jeong BH, Kim YS, Jang MK (2009). Association of concurrent hepatitis B surface antigen and antibody to hepatitis B surface antigen with hepatocellular carcinoma in chronic hepatitis B virus infection. J Med Virol.

[8] Seo SI, Choi HS, Choi BY, Kim HS, Kim HY, Jang MK (2014). Coexistence of hepatitis B surface antigen and antibody to hepatitis B surface may increase the risk of hepatocellular carcinoma in chronic hepatitis B virus infection: a retrospective cohort study. J Med Virol.

[9] Chen Y, Qian F, Yuan Q, Li X, Wu W, Guo X, Li L (2011). Mutations in hepatitis B virus DNA from patients with coexisting HBsAg/anti-HBs. J Clin Virol.

[10] Wu C, Zhang X, Tian Y, Song J, Yang D, Roggendorf M, Lu M, Chen X (2010). Biological significance of amino acid substitutions in hepatitis B surface antigen (HBsAg) for glycosylation, secretion, antigenicity and immunogenicity of HBsAg and hepatitis B virus replication. J Gen Virol.

[11] Julithe R, Aboujaoudé G, Sureau C (2014). Modification of the hepatitis B virus envelope protein glycosylation pattern interferes with secretion of viral particles, infectivity, and susceptibility to neutralizing antibodies. J Virol.

[12] Yu DM, Li XH, Mom V, Lu ZH, Liao XW, Han Y, Pichoud C, Gong QM, Zhang DH, Zhang Y, Deny P, Zoulim F, Zhang XX (2014). N-glycosylation mutations within hepatitis B virus surface major hydrophilic region contribute mostly to immune escape. J Hepatol.

[13] Salpini R, Colagrossi L, Bellocchi MC, Surdo M, Becker C, Alteri C, Aragri M, Ricciardi A, Armenia D, Pollicita M, Di Santo F, Carioti L, Louzoun Y (2015). Hepatitis B surface antigen genetic elements critical for immune escape correlate with hepatitis B virus reactivation upon immunosuppression. Hepatology.

[14] Pollicino T, Cacciola I, Saffioti F, Raimondo G (2014). Hepatitis B virus PreS/S gene variants: pathobiology and clinical implications. J Hepatol.

[15] Liu W, Cao Y, Wang T, Xiang G, Lu J, Zhang J, Hou P (2013). The N-glycosylation modification of LHBs (Large Surface Proteins of HBV) effects on endoplasmic reticulum stress, cell proliferation and its secretion. Hepat Mon.

[16] Chen J, Liu Y, Zhao J, Xu Z, Chen R, Si L, Lu S, Li X, Wang S, Zhang K, Li J, Han J, Xu D (2016). Characterization of novel Hepatitis B virus preS/S-gene mutations from a patient with occult HBV infection. PLoS One.

[17] Guo X, Jin Y, Qian G, Tu H (2008). Sequential accumulation of the mutations in core promoter of hepatitis B virus is associated with the development of hepatocellular carcinoma in Qidong, China. J Hepatol.

[18] Qu L, Kuai X, Liu T, Chen T, Ni Z, Shen X (2013). Pre-S deletion and complex mutations of hepatitis B virus related to young age hepatocellular carcinoma in Qidong, China. PLoS One.

[19] Chinese Society of Infectious Diseases and Parasitology, Chinese Society of Hepatology (2005). [The guideline of prevention and treatment for chronic hepatitis B]. [Article in Chinese]. Chin J Infec Dis.

[20] Liver Failure and Artificial Liver Group, Chinese Society of Infectious Diseases and Parasitology, Severe Liver Diseases and Artificial Liver Group, Chinese Society of Hepatology (2006). [Diagnostic and treatment guidelines for liver failure]. [Article in Chinese]. J Clin Hepatol (Chinese).

[21] Liu Y, Zhong Y, Zou Z, Xu Z, Li B, Ren X, Bai S, Wang L, Li X, Dai J, Wang Y, Mao P, Xu D (2010). Features and clinical implications of hepatitis B virus genotypes and mutations in basal core promoter/precore region in 507 Chinese patients with acute and chronic hepatitis B.. J Clin Virol.

[22] Xu Z, Ren X, Liu Y, Li X, Bai S, Zhong Y, Wang L, Mao P, Wang H, Xin S, Wong VW, Chan HL, Zoulim F, Xu D (2011). Association of hepatitis B virus mutations in basal core promoter and precore regions with severity of liver disease: an investigation of 793 Chinese patients with mild and severe chronic hepatitis B and acute-on-chronic liver failure. J Gastroenterol.

[23] Liu Y, Wang C, Zhong Y, Li X, Dai J, Ren X, Xu Z, Li L, Yao Z, Ji D, Wang L, Zhang L, Zoulim F, Xu D (2011). Genotypic resistance profile of hepatitis B virus (HBV) in a large cohort of nucleos(t)ide analog(s)-experienced Chinese patients with chronic HBV infection. J Viral Hepat.

[24] Ren X, Xu Z, Liu Y, Li X, Bai S, Ding N, Zhong Y, Wnag L, Mao P, Xu D (2010). HBV genotype and basal core promoter/precore mutations are associated with hepatitis B-related acute-on-chronic liver failure without preexisting liver cirrhosis. J Viral Hepat.

[25] Li X, Wang L, Zhong Y, Wong VW, Xu Z, Liu Y, Li Q, Xing S, Zhao J, Xu D (2010). Hepatitis B virus (HBV) subgenotypes C2 and B2 differ in lamivudine- and adefovir-resistance-associated mutational patterns in HBV-infected Chinese patients. J Clin Microbiol.

[26] Liu Y, Li X, Xin S, Xu Z, Chen R, Yang J, Liu L, Wong VW, Yang D, Chan HL, Xu D (2015). The rtA181S mutation of hepatitis B virus primarily confers resistance to adefovir dipivoxil. J Viral Hepat.

[27] Ji D, Liu Y, Li L, Xu Z, Si LL, Dai JZ, Li X, Wang L, Yao Z, Xin SJ, Chen GF, Xu D (2012). The rtL229 substitutions in the reverse transcriptase region of hepatitis B virus (HBV) polymerase are potentially associated with lamivudine resistance as a compensatory mutation. J Clin Virol.

[28] Xiong X, Sun D, Chai H, Shan W, Yu Y, Pu L, Cheng F (2015). MiR-145 functions as a tumor suppressor targeting NUAK1 in human intrahepatic cholangiocarcinoma. Biochem Biophys Res Commun.

[29] Deng G, Zhu L, Huang F, Nie W, Huang W, Xu H, Zheng S, Yi Z, Wan T (2015). SALL4 is a novel therapeutic target in intrahepatic cholangiocarcinoma. Oncotarget.

