# Supplementary material for: Additional N-glycosylation mutation in the major hydrophilic region of hepatitis B virus S gene is a risk indicator for hepatocellular carcinoma occurrence in patients with coexistence of HBsAg/anti-HBs
Source: Oncotarget. 2017 Jun 27;8(37):61719–30. doi: 10.18632/oncotarget.18682 (PMC5617459; doi:10.18632/oncotarget.18682)
Supplement: Supplementary file 1 [file oncotarget-08-61719-s001.pdf]

Additional N-glycosylation mutation in the major hydrophilic region of hepatitis B virus S gene is a risk indicator for hepatocellular carcinoma occurrence in patients with coexistence of HBsAg/anti-HBs

SUPPLEMENTARY MATERIALS

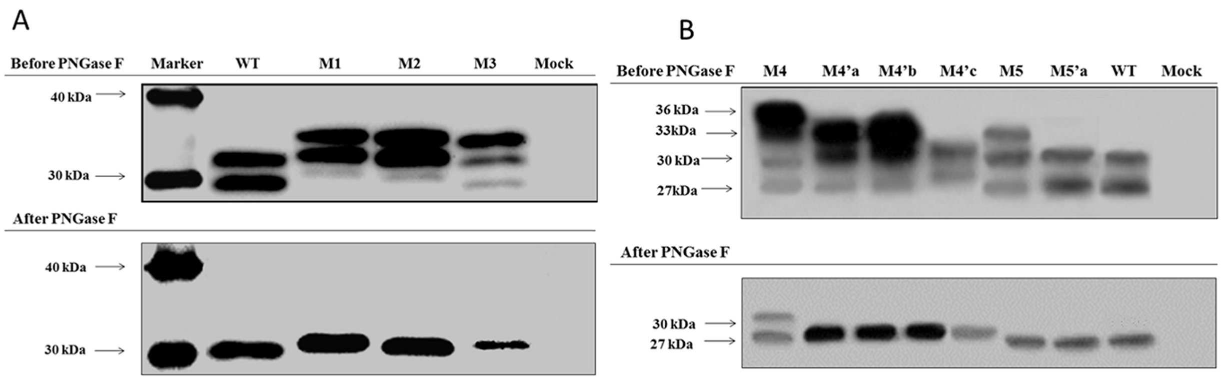

Supplementary Figure 1: Confirmation of additional N-glycosylation mutations at translational level. Samples were incubated with PNGase F, then analyzed by Western blotting with anti-His-tag antibody. **(A)** Comparison of key amino acid sequences between the wild-type and three mutants with single N-glycosylation mutation. WT, wild-type; M1, 115-116"INGTST" insertion; M2, 114-115"NTSTT" insertion; M3, 112-113"KNA" insertion→114-116NAS. **(B)** Comparison of key amino acid sequences between the wild-type and two mutants with single or dual N-glycosylation mutation(s), as well as their de-gycosylation counterparts (M4'a, M4'b, M4'c, M5'a) generated by reverse site-directed mutagenesis. M4, sT116N→116-118NST &sT131N+M133T→131-133NST; M4'a, sT116N→116-118NST; M4'b, sT131N+M133T→131-133NST; M4'c, artificially-generated wild-type from M4'a; M5, sG130N+T131S→130-132NSS; M5'a, artificially-generated wild-type from M5. Each additional N-glycosylation mutation adds approximately 3-kDa increased molecular weight.

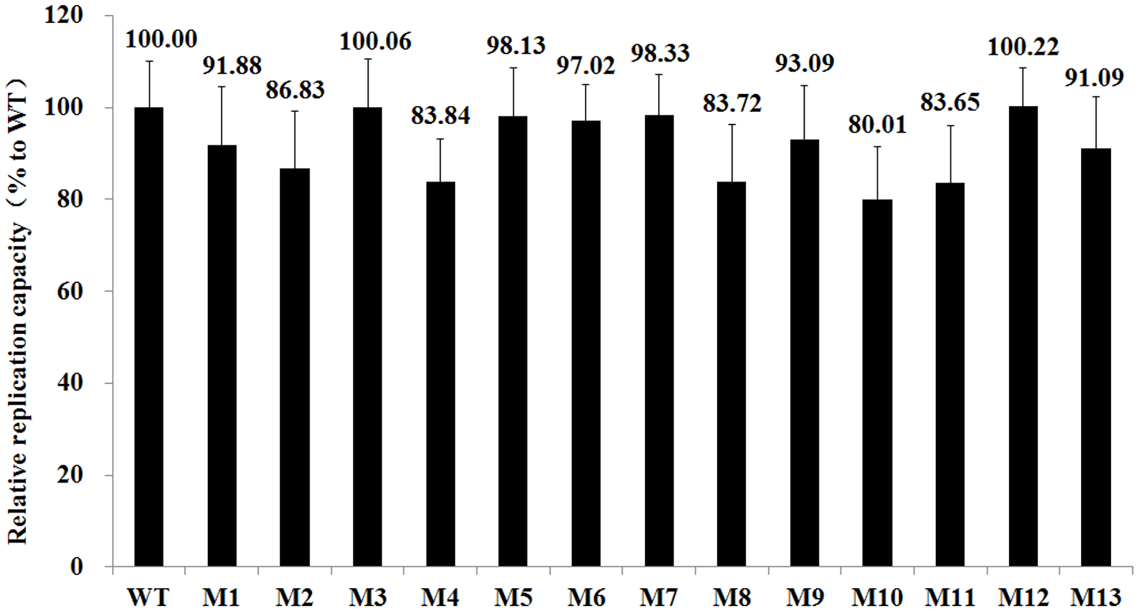

Supplementary Figure 2: Measurement of HBV replicative intermediates. WT, wild-type; M1, 115-116"NGTST" insertion→117-119NGT; M2, 112-113"KNA" insertion→114-116NAS; M3, 114-115"NTSTT" insertion→115-117NTS; M4, sT116N→116-118NST & sT131N+M133T→131-133NST; M5, sT116N→116-118NST; M6, sT131N+M133T→131-133NST; M7, sT113N+114–116"STT" deletion→113–115NST & sT131N+M133T→131-133NST; M8, sQ129N →129-131NGT; M9, 114-115 "TTN" insertion→117-119NST; M10, sG130N→130-132NTS; M11, sG130N+T131I →130-132NIS; M12, sG130N+T131N→130-132NNS; M13, sT113N → 113-115NST. The experiments were performed at least three times independently. Means ± standard deviations are presented. The difference in the replicative intermediate levels between each mutant and the wild-type was not statistically significant (P all > 0.05).

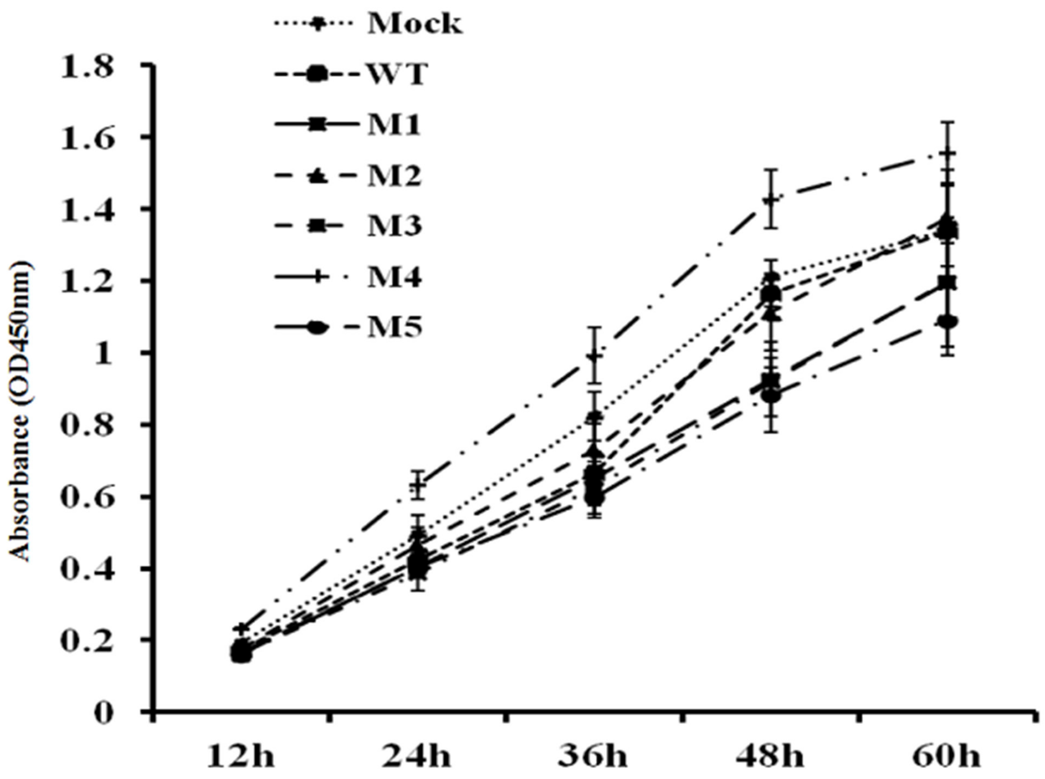

Supplementary Figure 3: Cell proliferation assay. WT, wild-type; M1, 115-116"INGTST" insertion→117–119NGT; M2, 114-115"NTSTT" insertion→115–117NTS; M3, 112-113"KNA" insertion→114-116NAS; M4, sT116N→116-118NST & sT131N+M133T→131-133NST; M5, sT131N+M133T→131-133NST. The experiments were performed at least three times independently. Means ± standard deviations are presented. The difference in the replicative intermediate levels between each mutant and the wild-type was not statistically significant (P all > 0.05).

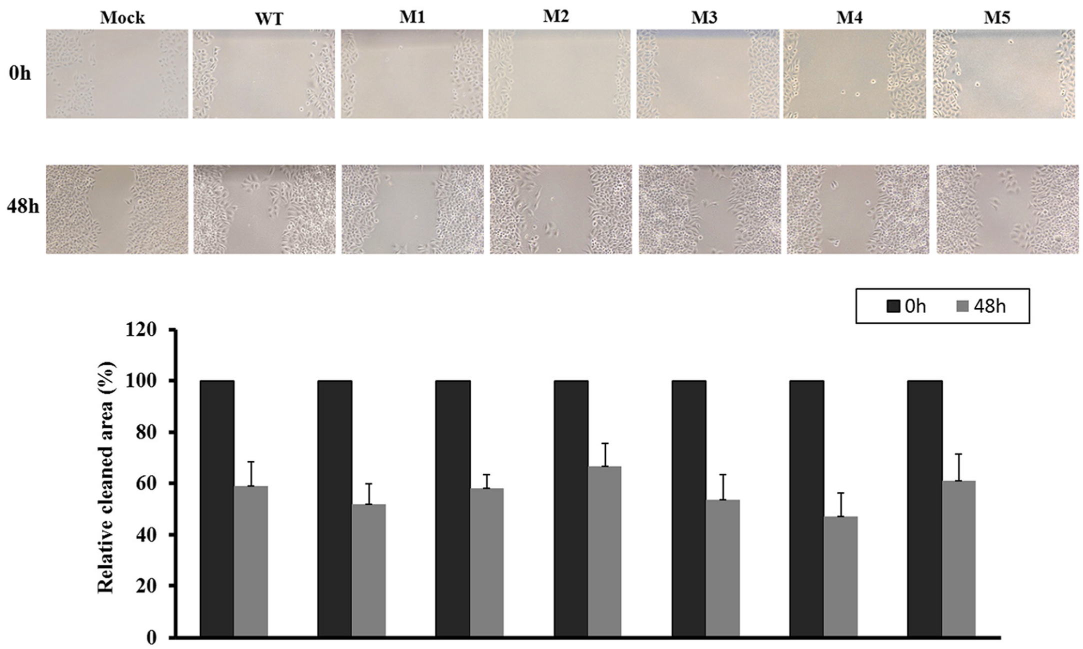

Supplementary Figure 4: Wound healing assay. WT, wild-type; M1, 115-116"INGTST" insertion→117–119NGT; M2, 114-115"NTSTT" insertion→115–117NTS; M3, 112-113"KNA" insertion→114-116NAS; M4, sT116N→116-118NST & sT131N+M133T→131-133NST; M5, sT131N+M133T→131-133NST. At least six microscopy images were examined for each sample and the experiments were performed twice. Results are representative of two similar experiments. Means ± standard deviations are presented. The difference in the replicative intermediate levels between each mutant and the wild-type was not statistically significant (*P* all > 0.05).

Supplementary Table 1: Analysis of additional N-glycosylation mutation frequencies in different illness categories based on age stratification

Subgroup		HCC (n = 38)	CHB (n = 230)	ACLF (n = 77)	*P* value
30-46 (n = 345)	Average age	39.50 ± 4.39	38.07 ± 5.50	38.12 ± 5.50	0.312
	N-glycosylation mutation frequency	6 (15.79%)	9 (3.91%)	2 (2.60%)	0.004
		HCC (n = 116)	CHB (n = 121)	ACLF (n = 59)	
47-66 (n = 296)	Average age	55.56 ± 5.09	54.31 ± 4.61	54.69 ± 4.55	0.127
	N-glycosylation mutation frequency	14 (12.39%)	6 (4.96%)	2 (3.39%)	0.047

HCC, hepatocellular carcinoma; CHB, chronic hepatitis B; ACLF, acute-on-chronic liver failure.

Supplementary Table 2: Comparison of ALT levels between HCC and non-HCC patients

	Diagnosis	With N-glycosylation mutations	Without N-glycosylation mutations	*P* value
ALT levels in total patients (U/L)	HCC	45.00 (32.25-5750)	47.00 (30.00-87.00)	0.426
	Non-HCC	67.00 (27.00-146.00)	60.00 (29.75-142.25)	0.163
ALT levels in coexistent HBsAg/antiHBs patients (U/L)	HCC	39.00 (32.00-57.00)	39.00 (26.00-72.50)	0.782
	Non-HCC	61.00 (27.00-132.00)	60.00 (30.50-142.50)	0.811

ALT, alanine aminotransferase; HCC, hepatocellular carcinoma.
